# Bis[μ-2,2′-dimethyl-1,1′-(3-oxapentane-1,5-di­yl)di-1*H*-benzimidazole-κ^2^ *N* ^3^:*N* ^3′^]bis­[bis­(4-meth­oxy­benzoato)-κ*O*;κ^2^ *O*,*O*′-cobalt(II)]

**DOI:** 10.1107/S160053681004568X

**Published:** 2010-11-13

**Authors:** Lian-Peng Zhao

**Affiliations:** a BoHai University, JinZhou, LiaoNing, 121013, People’s Republic of China

## Abstract

The complete mol­ecule of the title complex, [Co_2_(C_8_H_7_O_3_)_4_(C_20_H_22_N_4_O)_2_], is a dimer of the paddle-wheel-type generated by crystallographic inversion symmetry. The Co^II^ ion is penta­coordinated by three O atoms from two 4-meth­oxy­benzoate anions (one bidentate and one monodentate) and two N atoms from two 2,2′-bis­(2-methyl-1*H*-benzimidazole)­ether ligands. This results in a very distorted trigonal–bipyramidal geometry for the metal ion, with both N atoms in equatorial sites. The dihedral angle between the benzimidazole ring systems in the ligand is 60.04 (8)°. The configuration of the mol­ecule is supported by intra­molecular C—H⋯O hydrogen bonds.

## Related literature

For background to benzimidazole ligands in coordination polymers, see: Hoskins *et al.* (1997 ▶). For a related structure, see: Dimitrou *et al.* (1999 ▶).
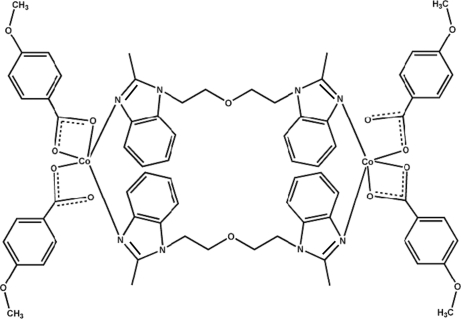


## Experimental

### 

#### Crystal data

[Co_2_(C_8_H_7_O_3_)_4_(C_20_H_22_N_4_O)_2_]
*M* *_r_* = 1391.24
Monoclinic, 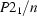

*a* = 12.4127 (3) Å
*b* = 16.3933 (4) Å
*c* = 17.6106 (5) Å
β = 109.577 (3)°
*V* = 3376.34 (15) Å^3^
*Z* = 2
Mo *K*α radiation
μ = 0.56 mm^−1^
*T* = 293 K
0.32 × 0.28 × 0.24 mm

#### Data collection

Oxford Diffraction Gemini R Ultra CCD diffractometer
Absorption correction: multi-scan (*CrysAlis RED*; Oxford Diffraction, 2006 ▶) *T*_min_ = 0.829, *T*_max_ = 0.889
14632 measured reflections
7801 independent reflections
3677 reflections with *I* > 2σ(*I*)
*R*_int_ = 0.030

#### Refinement

*R*[*F* ^2^ > 2σ(*F* ^2^)] = 0.038
*wR*(*F* ^2^) = 0.081
*S* = 0.75
7801 reflections
433 parameters
H-atom parameters constrained
Δρ_max_ = 0.48 e Å^−3^
Δρ_min_ = −0.24 e Å^−3^

### 

Data collection: *CrysAlis CCD* (Oxford Diffraction, 2006 ▶); cell refinement: *CrysAlis CCD*; data reduction: *CrysAlis RED* (Oxford Diffraction, 2006 ▶); program(s) used to solve structure: *SHELXS97* (Sheldrick, 2008 ▶); program(s) used to refine structure: *SHELXL97* (Sheldrick, 2008 ▶); molecular graphics: *SHELXTL* (Sheldrick, 2008 ▶); software used to prepare material for publication: *SHELXL97*.

## Supplementary Material

Crystal structure: contains datablocks I, global. DOI: 10.1107/S160053681004568X/hb5721sup1.cif

Structure factors: contains datablocks I. DOI: 10.1107/S160053681004568X/hb5721Isup2.hkl

Additional supplementary materials:  crystallographic information; 3D view; checkCIF report

## Figures and Tables

**Table 1:** Selected bond lengths (Å)

Co1—O6	1.9694 (14)
Co1—O3	2.0362 (18)
Co1—N1	2.0524 (18)
Co1—N4^i^	2.0598 (18)
Co1—O2	2.374 (2)

Symmetry code: (i) 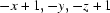.

**Table 2:** Hydrogen-bond geometry (Å, °)

*D*—H⋯*A*	*D*—H	H⋯*A*	*D*⋯*A*	*D*—H⋯*A*
C17—H015⋯O6^i^	0.93	2.46	3.111 (3)	127
C8—H8*C*⋯O2	0.96	2.50	3.255 (3)	136
C20—H03*B*⋯O2^i^	0.96	2.42	3.150 (3)	132

Symmetry code: (i) 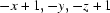.

**Fig. 1.**
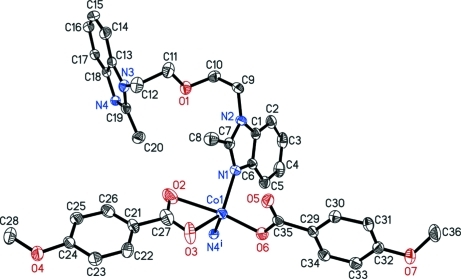
The molecular structure of the title compound with the atom numbering scheme. Displacement ellipsoids are drawn at the 30% probability level. H atoms have been omitted for clarity. [symmetry code: (i) - x + 1, - y, - z + 1.]

**Fig. 2.**
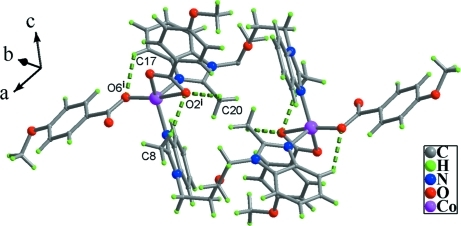
C—H···O interactions (dotted lines) in the title compound.: [symmetry code: (i) -x + 1, -y, -z + 1.]

## supplementary crystallographic information

### Comment

Bis(imidazole) ligands with –CH_2_– spacers are a good candidate for N-donor bridging ligand (Hoskins *et al.*, 1997). Up to now, 2,2'-bis(2-methyl-1*H*-benzimidazole)ether ligands, as a flexible ligand, is rarely investigated in constructing coordination polymers.

In the title compound (Fig. 1), the Co^II^ ion is pentacoordinated by three O atoms from two 4-methoxybenzoate anions, and two N atoms from two 2,2'-bis(2-methyl-1*H*-benzimidazole)ether ligand. The Co—O distances are found in the range from 1.9694 (14) to 2.374 (2) Å, which is similar to previous report (Dimitrou *et al.*, 1999). The Co—N distances are 2.0524 (18) and 2.0598 (18) Å, respectively. The crystal structure is stabilized by a weak intermolecular C—H···O hydrogen bond between the benzene H atom of 2-methyl-1*H*-benzimidazole ring and the O atom of diethyl ether group, with a C17—H015···O6; the methyl H atoms of 2-methyl-1*H*-benzimidazole ligands and the carboxylate O atoms with C8—H8c—O2 and C20—H03B—O2 (Table 1 & Fig. 2).

### Experimental

An aqueous solution (10 ml) of 4-methoxybenzoic acid (0.072 g, 0.4 mmol), 2,2'-bis(2-methylbenzimidazole)ether (0.065 g, 0.2 mmol) and Co(Ac)_2_ (0.049 g, 0.2 mmol) was added in and sealed in 18 ml Teflon-lined stainless steel container. The container was heated to 130 °C and held at that temperature for 72 h, then cooled to room temperature at a rate of 10 °C.h^-1^. And then the title compound was isolated as purple blocks.

### Refinement

C–bound H–atoms were geometrically positioned (C—H 0.93 Å) and refined using a riding model, with *U*_iso_ = 1.2*U*_eq_ (C).

### Crystal data

**Table:**

[Co_2_(C_8_H_7_O_3_)_4_(C_20_H_22_N_4_O)_2_]	*F*(000) = 1452
*M**_r_* = 1391.24	*D*_x_ = 1.368 Mg m^−^^3^
Monoclinic, *P*2_1_/*n*	Mo *K*α radiation, λ = 0.71073 Å
Hall symbol: -P 2yn	Cell parameters from 7801 reflections
*a* = 12.4127 (3) Å	θ = 3.0–29.2°
*b* = 16.3933 (4) Å	µ = 0.56 mm^−^^1^
*c* = 17.6106 (5) Å	*T* = 293 K
β = 109.577 (3)°	Block, purple
*V* = 3376.34 (15) Å^3^	0.32 × 0.28 × 0.24 mm
*Z* = 2	

### Data collection

**Table:**

Oxford Diffraction Gemini R Ultra CCD diffractometer	7801 independent reflections
Radiation source: fine-focus sealed tube	3677 reflections with *I* > 2σ(*I*)
graphite	*R*_int_ = 0.030
Detector resolution: 10.0 pixels mm^-1^	θ_max_ = 29.2°, θ_min_ = 3.1°
ω scans	*h* = −15→17
Absorption correction: multi-scan (*CrysAlis RED*; Oxford Diffraction, 2006)	*k* = −21→22
*T*_min_ = 0.829, *T*_max_ = 0.889	*l* = −23→23
14632 measured reflections	

### Refinement

**Table:**

Refinement on *F*^2^	Primary atom site location: structure-invariant direct methods
Least-squares matrix: full	Secondary atom site location: difference Fourier map
*R*[*F*^2^ > 2σ(*F*^2^)] = 0.038	Hydrogen site location: inferred from neighbouring sites
*wR*(*F*^2^) = 0.081	H-atom parameters constrained
*S* = 0.75	*w* = 1/[σ^2^(*F*_o_^2^) + (0.0363*P*)^2^] where *P* = (*F*_o_^2^ + 2*F*_c_^2^)/3
7801 reflections	(Δ/σ)_max_ = 0.002
433 parameters	Δρ_max_ = 0.48 e Å^−^^3^
0 restraints	Δρ_min_ = −0.24 e Å^−^^3^

### Special details

**Table:**

Geometry. All s.u.'s (except the s.u. in the dihedral angle between two l.s. planes) are estimated using the full covariance matrix. The cell s.u.'s are taken into account individually in the estimation of s.u.'s in distances, angles and torsion angles; correlations between s.u.'s in cell parameters are only used when they are defined by crystal symmetry. An approximate (isotropic) treatment of cell s.u.'s is used for estimating s.u.'s involving l.s. planes.
Refinement. Refinement of *F*^2^ against ALL reflections. The weighted *R*-factor *wR* and goodness of fit *S* are based on *F*^2^, conventional *R*-factors *R* are based on *F*, with *F* set to zero for negative *F*^2^. The threshold expression of *F*^2^ > 2σ(*F*^2^) is used only for calculating *R*-factors(gt) *etc*. and is not relevant to the choice of reflections for refinement. *R*-factors based on *F*^2^ are statistically about twice as large as those based on *F*, and *R*- factors based on ALL data will be even larger.

### Fractional atomic coordinates and isotropic or equivalent isotropic displacement parameters (Å^2^)

**Table:**

	*x*	*y*	*z*	*U*_iso_*/*U*_eq_	
Co1	0.78779 (2)	0.131671 (18)	0.654674 (17)	0.03902 (10)	
C1	0.85504 (17)	−0.01846 (14)	0.47885 (14)	0.0403 (6)	
C2	0.89543 (19)	−0.09166 (17)	0.45938 (17)	0.0565 (7)	
H030	0.8957	−0.1022	0.4076	0.068*	
C3	0.9350 (2)	−0.14773 (16)	0.5202 (2)	0.0657 (8)	
H026	0.9626	−0.1976	0.5094	0.079*	
C4	0.9347 (2)	−0.13176 (17)	0.59687 (19)	0.0636 (8)	
H046	0.9621	−0.1713	0.6365	0.076*	
C5	0.89485 (18)	−0.05868 (15)	0.61701 (15)	0.0495 (6)	
H019	0.8951	−0.0483	0.6690	0.059*	
C6	0.85452 (17)	−0.00168 (13)	0.55553 (13)	0.0364 (5)	
C7	0.78846 (17)	0.10552 (13)	0.48290 (13)	0.0386 (6)	
C8	0.7453 (2)	0.18927 (14)	0.45810 (14)	0.0581 (7)	
H8A	0.7340	0.1966	0.4019	0.087*	
H8B	0.8000	0.2284	0.4891	0.087*	
H8C	0.6740	0.1969	0.4673	0.087*	
C9	0.79201 (19)	0.05843 (17)	0.34709 (13)	0.0538 (7)	
H9A	0.8564	0.0359	0.3346	0.065*	
H9B	0.7852	0.1156	0.3322	0.065*	
C10	0.68383 (19)	0.01401 (16)	0.29879 (14)	0.0551 (7)	
H10A	0.6757	0.0162	0.2421	0.066*	
H10B	0.6902	−0.0429	0.3149	0.066*	
C11	0.53616 (19)	0.11094 (16)	0.25483 (16)	0.0604 (8)	
H11A	0.5161	0.0914	0.1998	0.073*	
H11B	0.5895	0.1558	0.2621	0.073*	
C12	0.4298 (2)	0.13882 (15)	0.27170 (16)	0.0590 (7)	
H12A	0.4511	0.1590	0.3266	0.071*	
H12B	0.3942	0.1832	0.2356	0.071*	
C13	0.27905 (18)	0.04282 (15)	0.18685 (14)	0.0425 (6)	
C14	0.2593 (2)	0.07031 (17)	0.10893 (16)	0.0606 (7)	
H047	0.2954	0.1164	0.0982	0.073*	
C15	0.1834 (2)	0.0255 (2)	0.04845 (16)	0.0692 (8)	
H031	0.1678	0.0419	−0.0047	0.083*	
C16	0.1298 (2)	−0.04286 (19)	0.06417 (15)	0.0614 (8)	
H037	0.0792	−0.0713	0.0213	0.074*	
C17	0.14893 (18)	−0.07034 (15)	0.14168 (13)	0.0476 (6)	
H015	0.1121	−0.1163	0.1520	0.057*	
C18	0.22604 (17)	−0.02586 (14)	0.20371 (12)	0.0376 (5)	
C19	0.33864 (17)	0.02086 (14)	0.31804 (14)	0.0419 (6)	
C20	0.4034 (2)	0.03192 (16)	0.40569 (13)	0.0584 (7)	
H03A	0.4512	0.0793	0.4131	0.088*	
H03B	0.4500	−0.0153	0.4258	0.088*	
H03C	0.3505	0.0388	0.4344	0.088*	
C21	0.50942 (19)	0.27294 (15)	0.61817 (15)	0.0480 (6)	
C22	0.52284 (19)	0.34163 (15)	0.66575 (15)	0.0516 (7)	
H024	0.5952	0.3554	0.7006	0.062*	
C23	0.43083 (19)	0.39010 (15)	0.66240 (14)	0.0504 (6)	
H029	0.4414	0.4364	0.6946	0.061*	
C24	0.32260 (17)	0.36994 (15)	0.61112 (13)	0.0423 (6)	
C25	0.3085 (2)	0.30181 (15)	0.56284 (14)	0.0490 (6)	
H034	0.2364	0.2880	0.5276	0.059*	
C26	0.4016 (2)	0.25449 (15)	0.56708 (14)	0.0541 (7)	
H023	0.3913	0.2086	0.5344	0.065*	
C27	0.6097 (3)	0.21856 (18)	0.6219 (2)	0.0646 (8)	
C28	0.1242 (2)	0.40712 (18)	0.55740 (16)	0.0730 (9)	
H04A	0.0725	0.4468	0.5658	0.109*	
H04B	0.0988	0.3534	0.5650	0.109*	
H04C	0.1260	0.4121	0.5035	0.109*	
C29	1.13722 (17)	0.17700 (13)	0.74857 (13)	0.0355 (5)	
C30	1.21576 (17)	0.21342 (13)	0.71996 (13)	0.0408 (6)	
H033	1.1897	0.2450	0.6735	0.049*	
C31	1.33219 (17)	0.20458 (14)	0.75815 (14)	0.0448 (6)	
H022	1.3837	0.2289	0.7370	0.054*	
C32	1.37090 (19)	0.15926 (14)	0.82796 (14)	0.0447 (6)	
C33	1.29372 (19)	0.12518 (15)	0.85978 (14)	0.0488 (6)	
H032	1.3200	0.0970	0.9083	0.059*	
C34	1.17833 (18)	0.13266 (14)	0.82017 (13)	0.0439 (6)	
H014	1.1271	0.1080	0.8412	0.053*	
C35	1.01124 (18)	0.18316 (15)	0.70171 (14)	0.0412 (6)	
C36	1.5609 (2)	0.14637 (19)	0.82543 (18)	0.0758 (9)	
H04D	1.6368	0.1350	0.8612	0.114*	
H04E	1.5593	0.1994	0.8019	0.114*	
H04F	1.5389	0.1060	0.7835	0.114*	
N1	0.81165 (14)	0.07684 (10)	0.55674 (10)	0.0370 (4)	
N2	0.81263 (14)	0.05087 (12)	0.43346 (10)	0.0406 (5)	
N3	0.34858 (14)	0.07201 (11)	0.26072 (11)	0.0447 (5)	
N4	0.26514 (13)	−0.03898 (11)	0.28711 (10)	0.0380 (4)	
O1	0.58565 (13)	0.04771 (9)	0.30952 (9)	0.0513 (4)	
O2	0.59500 (18)	0.15921 (14)	0.57565 (13)	0.0890 (7)	
O3	0.70491 (15)	0.23292 (11)	0.67326 (14)	0.0804 (6)	
O4	0.23632 (13)	0.42058 (10)	0.61380 (10)	0.0609 (5)	
O5	0.97677 (12)	0.22669 (10)	0.64168 (10)	0.0526 (4)	
O6	0.94624 (12)	0.14002 (11)	0.72914 (9)	0.0554 (5)	
O7	1.48382 (13)	0.14450 (12)	0.86905 (10)	0.0731 (6)	

### Atomic displacement parameters (Å^2^)

**Table:**

	*U*^11^	*U*^22^	*U*^33^	*U*^12^	*U*^13^	*U*^23^
Co1	0.03659 (16)	0.04231 (18)	0.04015 (18)	−0.00346 (15)	0.01550 (13)	−0.00498 (16)
C1	0.0299 (11)	0.0410 (15)	0.0488 (15)	−0.0039 (11)	0.0118 (11)	−0.0119 (12)
C2	0.0386 (13)	0.0638 (18)	0.0658 (18)	−0.0066 (13)	0.0158 (13)	−0.0274 (16)
C3	0.0438 (14)	0.0472 (18)	0.100 (2)	0.0032 (13)	0.0161 (16)	−0.0228 (18)
C4	0.0500 (15)	0.0445 (16)	0.091 (2)	0.0081 (14)	0.0161 (15)	0.0103 (16)
C5	0.0454 (13)	0.0489 (16)	0.0532 (15)	0.0048 (12)	0.0154 (12)	0.0050 (13)
C6	0.0323 (11)	0.0365 (14)	0.0418 (14)	−0.0036 (10)	0.0143 (10)	−0.0048 (11)
C7	0.0377 (12)	0.0388 (14)	0.0414 (14)	−0.0060 (10)	0.0159 (11)	0.0002 (11)
C8	0.0724 (17)	0.0465 (16)	0.0559 (16)	0.0027 (14)	0.0223 (13)	0.0081 (13)
C9	0.0452 (13)	0.0805 (19)	0.0418 (15)	−0.0028 (13)	0.0226 (12)	−0.0048 (13)
C10	0.0534 (15)	0.0639 (18)	0.0432 (15)	0.0025 (14)	0.0099 (12)	−0.0089 (13)
C11	0.0445 (14)	0.0584 (18)	0.0762 (18)	−0.0063 (13)	0.0174 (13)	0.0259 (15)
C12	0.0530 (15)	0.0412 (15)	0.0800 (19)	−0.0060 (13)	0.0188 (13)	0.0124 (14)
C13	0.0346 (12)	0.0507 (15)	0.0443 (15)	0.0092 (11)	0.0158 (11)	0.0094 (12)
C14	0.0545 (15)	0.0720 (19)	0.0570 (17)	0.0119 (15)	0.0208 (14)	0.0236 (15)
C15	0.0663 (18)	0.102 (2)	0.0412 (16)	0.0232 (18)	0.0205 (15)	0.0208 (16)
C16	0.0471 (15)	0.094 (2)	0.0393 (16)	0.0109 (16)	0.0097 (13)	−0.0081 (15)
C17	0.0373 (12)	0.0633 (17)	0.0426 (14)	0.0018 (12)	0.0139 (11)	−0.0062 (13)
C18	0.0319 (11)	0.0471 (14)	0.0352 (13)	0.0068 (11)	0.0131 (10)	0.0011 (11)
C19	0.0325 (12)	0.0476 (15)	0.0476 (14)	−0.0042 (11)	0.0161 (11)	−0.0021 (12)
C20	0.0495 (14)	0.0718 (19)	0.0480 (15)	−0.0159 (13)	0.0086 (12)	−0.0107 (13)
C21	0.0462 (14)	0.0453 (16)	0.0622 (17)	0.0049 (12)	0.0312 (13)	0.0082 (13)
C22	0.0394 (13)	0.0496 (16)	0.0640 (17)	−0.0047 (12)	0.0147 (12)	0.0030 (13)
C23	0.0496 (14)	0.0457 (15)	0.0547 (15)	−0.0037 (12)	0.0158 (12)	−0.0076 (12)
C24	0.0420 (13)	0.0437 (14)	0.0440 (13)	0.0039 (12)	0.0180 (11)	0.0003 (12)
C25	0.0478 (14)	0.0511 (16)	0.0468 (15)	−0.0040 (13)	0.0142 (12)	−0.0057 (13)
C26	0.0627 (17)	0.0464 (15)	0.0573 (16)	0.0002 (14)	0.0256 (14)	−0.0089 (13)
C27	0.068 (2)	0.0535 (19)	0.086 (2)	0.0103 (16)	0.0438 (18)	0.0239 (17)
C28	0.0494 (15)	0.085 (2)	0.0741 (19)	0.0162 (15)	0.0070 (14)	0.0024 (16)
C29	0.0339 (11)	0.0330 (12)	0.0432 (14)	−0.0032 (10)	0.0176 (10)	−0.0074 (11)
C30	0.0419 (13)	0.0370 (13)	0.0434 (13)	0.0013 (11)	0.0142 (11)	0.0033 (11)
C31	0.0358 (12)	0.0461 (15)	0.0564 (16)	−0.0041 (11)	0.0205 (11)	0.0006 (12)
C32	0.0379 (13)	0.0468 (15)	0.0476 (15)	0.0057 (11)	0.0120 (12)	−0.0079 (12)
C33	0.0539 (15)	0.0495 (15)	0.0432 (14)	0.0120 (13)	0.0165 (12)	0.0063 (12)
C34	0.0474 (13)	0.0411 (13)	0.0497 (14)	−0.0044 (12)	0.0246 (11)	−0.0009 (13)
C35	0.0394 (13)	0.0437 (15)	0.0446 (15)	−0.0020 (12)	0.0193 (12)	−0.0161 (13)
C36	0.0433 (14)	0.090 (2)	0.094 (2)	0.0140 (15)	0.0239 (15)	−0.0113 (18)
N1	0.0407 (10)	0.0347 (11)	0.0382 (11)	0.0001 (9)	0.0167 (8)	−0.0010 (9)
N2	0.0409 (10)	0.0501 (12)	0.0348 (11)	−0.0058 (9)	0.0178 (9)	−0.0049 (10)
N3	0.0380 (10)	0.0427 (12)	0.0528 (13)	−0.0033 (9)	0.0145 (10)	0.0080 (10)
N4	0.0326 (9)	0.0463 (12)	0.0356 (11)	−0.0060 (9)	0.0122 (8)	−0.0019 (9)
O1	0.0439 (9)	0.0492 (10)	0.0611 (11)	0.0013 (8)	0.0180 (8)	0.0112 (9)
O2	0.1034 (16)	0.0903 (17)	0.0858 (15)	0.0400 (13)	0.0482 (12)	−0.0019 (13)
O3	0.0442 (11)	0.0576 (12)	0.1416 (18)	0.0037 (9)	0.0341 (11)	0.0070 (12)
O4	0.0474 (9)	0.0658 (12)	0.0642 (11)	0.0129 (9)	0.0115 (9)	−0.0133 (9)
O5	0.0443 (9)	0.0537 (11)	0.0530 (11)	0.0047 (8)	0.0074 (8)	−0.0008 (9)
O6	0.0406 (9)	0.0787 (12)	0.0501 (10)	−0.0164 (9)	0.0196 (8)	−0.0075 (9)
O7	0.0366 (9)	0.1133 (17)	0.0645 (12)	0.0192 (10)	0.0106 (9)	0.0039 (11)

### Geometric parameters (Å, °)

**Table:**

Co1—O6	1.9694 (14)	C17—C18	1.393 (3)
Co1—O3	2.0362 (18)	C17—H015	0.9300
Co1—N1	2.0524 (18)	C18—N4	1.401 (3)
Co1—N4^i^	2.0598 (18)	C19—N4	1.326 (3)
Co1—O2	2.374 (2)	C19—N3	1.349 (3)
C1—C6	1.380 (3)	C19—C20	1.493 (3)
C1—C2	1.387 (3)	C20—H03A	0.9600
C1—N2	1.388 (3)	C20—H03B	0.9600
C2—C3	1.372 (4)	C20—H03C	0.9600
C2—H030	0.9300	C21—C26	1.373 (3)
C3—C4	1.377 (4)	C21—C22	1.380 (3)
C3—H026	0.9300	C21—C27	1.515 (4)
C4—C5	1.387 (3)	C22—C23	1.376 (3)
C4—H046	0.9300	C22—H024	0.9300
C5—C6	1.390 (3)	C23—C24	1.384 (3)
C5—H019	0.9300	C23—H029	0.9300
C6—N1	1.396 (3)	C24—O4	1.368 (3)
C7—N1	1.321 (3)	C24—C25	1.379 (3)
C7—N2	1.351 (3)	C25—C26	1.372 (3)
C7—C8	1.485 (3)	C25—H034	0.9300
C8—H8A	0.9600	C26—H023	0.9300
C8—H8B	0.9600	C27—O2	1.243 (3)
C8—H8C	0.9600	C27—O3	1.246 (3)
C9—N2	1.461 (3)	C28—O4	1.430 (3)
C9—C10	1.514 (3)	C28—H04A	0.9600
C9—H9A	0.9700	C28—H04B	0.9600
C9—H9B	0.9700	C28—H04C	0.9600
C10—O1	1.407 (3)	C29—C30	1.375 (3)
C10—H10A	0.9700	C29—C34	1.395 (3)
C10—H10B	0.9700	C29—C35	1.507 (3)
C11—O1	1.408 (3)	C30—C31	1.382 (3)
C11—C12	1.517 (3)	C30—H033	0.9300
C11—H11A	0.9700	C31—C32	1.378 (3)
C11—H11B	0.9700	C31—H022	0.9300
C12—N3	1.457 (3)	C32—O7	1.366 (2)
C12—H12A	0.9700	C32—C33	1.380 (3)
C12—H12B	0.9700	C33—C34	1.372 (3)
C13—N3	1.383 (3)	C33—H032	0.9300
C13—C18	1.386 (3)	C34—H014	0.9300
C13—C14	1.386 (3)	C35—O5	1.228 (3)
C14—C15	1.374 (4)	C35—O6	1.283 (3)
C14—H047	0.9300	C36—O7	1.414 (3)
C15—C16	1.379 (4)	C36—H04D	0.9600
C15—H031	0.9300	C36—H04E	0.9600
C16—C17	1.380 (3)	C36—H04F	0.9600
C16—H037	0.9300	N4—Co1^i^	2.0598 (18)
			
O6—Co1—O3	106.38 (8)	N4—C19—C20	124.9 (2)
O6—Co1—N1	101.35 (7)	N3—C19—C20	123.1 (2)
O3—Co1—N1	135.65 (8)	C19—C20—H03A	109.5
O6—Co1—N4^i^	97.69 (7)	C19—C20—H03B	109.5
O3—Co1—N4^i^	104.99 (8)	H03A—C20—H03B	109.5
N1—Co1—N4^i^	104.77 (7)	C19—C20—H03C	109.5
O6—Co1—O2	164.33 (8)	H03A—C20—H03C	109.5
O3—Co1—O2	58.32 (7)	H03B—C20—H03C	109.5
N1—Co1—O2	89.52 (7)	C26—C21—C22	118.1 (2)
N4^i^—Co1—O2	90.29 (7)	C26—C21—C27	120.4 (2)
C6—C1—C2	122.3 (2)	C22—C21—C27	121.6 (2)
C6—C1—N2	105.97 (19)	C23—C22—C21	121.0 (2)
C2—C1—N2	131.7 (2)	C23—C22—H024	119.5
C3—C2—C1	116.8 (3)	C21—C22—H024	119.5
C3—C2—H030	121.6	C22—C23—C24	120.0 (2)
C1—C2—H030	121.6	C22—C23—H029	120.0
C2—C3—C4	121.4 (3)	C24—C23—H029	120.0
C2—C3—H026	119.3	O4—C24—C25	125.03 (19)
C4—C3—H026	119.3	O4—C24—C23	115.6 (2)
C3—C4—C5	122.3 (3)	C25—C24—C23	119.4 (2)
C3—C4—H046	118.9	C26—C25—C24	119.6 (2)
C5—C4—H046	118.9	C26—C25—H034	120.2
C4—C5—C6	116.6 (2)	C24—C25—H034	120.2
C4—C5—H019	121.7	C25—C26—C21	121.9 (2)
C6—C5—H019	121.7	C25—C26—H023	119.0
C1—C6—C5	120.6 (2)	C21—C26—H023	119.0
C1—C6—N1	109.00 (19)	O2—C27—O3	121.3 (3)
C5—C6—N1	130.4 (2)	O2—C27—C21	119.6 (3)
N1—C7—N2	112.09 (19)	O3—C27—C21	119.1 (3)
N1—C7—C8	123.8 (2)	O4—C28—H04A	109.5
N2—C7—C8	124.0 (2)	O4—C28—H04B	109.5
C7—C8—H8A	109.5	H04A—C28—H04B	109.5
C7—C8—H8B	109.5	O4—C28—H04C	109.5
H8A—C8—H8B	109.5	H04A—C28—H04C	109.5
C7—C8—H8C	109.5	H04B—C28—H04C	109.5
H8A—C8—H8C	109.5	C30—C29—C34	117.89 (19)
H8B—C8—H8C	109.5	C30—C29—C35	120.2 (2)
N2—C9—C10	110.9 (2)	C34—C29—C35	121.9 (2)
N2—C9—H9A	109.5	C29—C30—C31	122.0 (2)
C10—C9—H9A	109.5	C29—C30—H033	119.0
N2—C9—H9B	109.5	C31—C30—H033	119.0
C10—C9—H9B	109.5	C32—C31—C30	119.1 (2)
H9A—C9—H9B	108.0	C32—C31—H022	120.5
O1—C10—C9	112.4 (2)	C30—C31—H022	120.5
O1—C10—H10A	109.1	O7—C32—C31	123.9 (2)
C9—C10—H10A	109.1	O7—C32—C33	116.2 (2)
O1—C10—H10B	109.1	C31—C32—C33	119.9 (2)
C9—C10—H10B	109.1	C34—C33—C32	120.4 (2)
H10A—C10—H10B	107.9	C34—C33—H032	119.8
O1—C11—C12	107.3 (2)	C32—C33—H032	119.8
O1—C11—H11A	110.3	C33—C34—C29	120.6 (2)
C12—C11—H11A	110.3	C33—C34—H014	119.7
O1—C11—H11B	110.3	C29—C34—H014	119.7
C12—C11—H11B	110.3	O5—C35—O6	124.3 (2)
H11A—C11—H11B	108.5	O5—C35—C29	120.6 (2)
N3—C12—C11	110.9 (2)	O6—C35—C29	115.1 (2)
N3—C12—H12A	109.5	O7—C36—H04D	109.5
C11—C12—H12A	109.5	O7—C36—H04E	109.5
N3—C12—H12B	109.5	H04D—C36—H04E	109.5
C11—C12—H12B	109.5	O7—C36—H04F	109.5
H12A—C12—H12B	108.1	H04D—C36—H04F	109.5
N3—C13—C18	105.72 (19)	H04E—C36—H04F	109.5
N3—C13—C14	131.8 (2)	C7—N1—C6	105.77 (18)
C18—C13—C14	122.5 (2)	C7—N1—Co1	128.77 (15)
C15—C14—C13	116.2 (3)	C6—N1—Co1	125.44 (14)
C15—C14—H047	121.9	C7—N2—C1	107.17 (18)
C13—C14—H047	121.9	C7—N2—C9	128.0 (2)
C14—C15—C16	122.0 (2)	C1—N2—C9	124.7 (2)
C14—C15—H031	119.0	C19—N3—C13	107.69 (18)
C16—C15—H031	119.0	C19—N3—C12	126.91 (18)
C15—C16—C17	122.0 (2)	C13—N3—C12	124.8 (2)
C15—C16—H037	119.0	C19—N4—C18	105.43 (18)
C17—C16—H037	119.0	C19—N4—Co1^i^	129.17 (15)
C16—C17—C18	116.7 (2)	C18—N4—Co1^i^	125.38 (14)
C16—C17—H015	121.6	C10—O1—C11	113.34 (19)
C18—C17—H015	121.6	C27—O2—Co1	82.33 (17)
C13—C18—C17	120.5 (2)	C27—O3—Co1	97.85 (18)
C13—C18—N4	109.08 (18)	C24—O4—C28	118.24 (18)
C17—C18—N4	130.4 (2)	C35—O6—Co1	113.66 (14)
N4—C19—N3	112.04 (19)	C32—O7—C36	118.0 (2)
			
C6—C1—C2—C3	0.3 (3)	O6—Co1—N1—C7	−117.02 (18)
N2—C1—C2—C3	178.1 (2)	O3—Co1—N1—C7	11.1 (2)
C1—C2—C3—C4	−0.1 (4)	N4^i^—Co1—N1—C7	141.80 (17)
C2—C3—C4—C5	−0.1 (4)	O2—Co1—N1—C7	51.62 (18)
C3—C4—C5—C6	0.2 (4)	O6—Co1—N1—C6	64.75 (17)
C2—C1—C6—C5	−0.2 (3)	O3—Co1—N1—C6	−167.17 (14)
N2—C1—C6—C5	−178.51 (18)	N4^i^—Co1—N1—C6	−36.44 (17)
C2—C1—C6—N1	178.64 (18)	O2—Co1—N1—C6	−126.61 (16)
N2—C1—C6—N1	0.3 (2)	N1—C7—N2—C1	−0.2 (2)
C4—C5—C6—C1	0.0 (3)	C8—C7—N2—C1	177.53 (19)
C4—C5—C6—N1	−178.6 (2)	N1—C7—N2—C9	175.86 (18)
N2—C9—C10—O1	63.5 (3)	C8—C7—N2—C9	−6.4 (3)
O1—C11—C12—N3	−60.1 (3)	C6—C1—N2—C7	−0.1 (2)
N3—C13—C14—C15	179.9 (2)	C2—C1—N2—C7	−178.2 (2)
C18—C13—C14—C15	0.1 (4)	C6—C1—N2—C9	−176.30 (18)
C13—C14—C15—C16	0.0 (4)	C2—C1—N2—C9	5.6 (3)
C14—C15—C16—C17	0.2 (4)	C10—C9—N2—C7	−98.9 (3)
C15—C16—C17—C18	−0.4 (4)	C10—C9—N2—C1	76.4 (3)
N3—C13—C18—C17	179.77 (19)	N4—C19—N3—C13	−1.5 (3)
C14—C13—C18—C17	−0.4 (3)	C20—C19—N3—C13	178.7 (2)
N3—C13—C18—N4	−1.2 (2)	N4—C19—N3—C12	−172.9 (2)
C14—C13—C18—N4	178.6 (2)	C20—C19—N3—C12	7.3 (4)
C16—C17—C18—C13	0.6 (3)	C18—C13—N3—C19	1.6 (2)
C16—C17—C18—N4	−178.3 (2)	C14—C13—N3—C19	−178.2 (3)
C26—C21—C22—C23	0.3 (4)	C18—C13—N3—C12	173.2 (2)
C27—C21—C22—C23	−179.3 (2)	C14—C13—N3—C12	−6.6 (4)
C21—C22—C23—C24	0.4 (4)	C11—C12—N3—C19	93.8 (3)
C22—C23—C24—O4	178.2 (2)	C11—C12—N3—C13	−76.3 (3)
C22—C23—C24—C25	−1.0 (4)	N3—C19—N4—C18	0.8 (2)
O4—C24—C25—C26	−178.3 (2)	C20—C19—N4—C18	−179.5 (2)
C23—C24—C25—C26	0.9 (4)	N3—C19—N4—Co1^i^	−177.77 (14)
C24—C25—C26—C21	−0.2 (4)	C20—C19—N4—Co1^i^	2.0 (3)
C22—C21—C26—C25	−0.4 (4)	C13—C18—N4—C19	0.3 (2)
C27—C21—C26—C25	179.2 (2)	C17—C18—N4—C19	179.2 (2)
C26—C21—C27—O2	3.5 (4)	C13—C18—N4—Co1^i^	178.91 (14)
C22—C21—C27—O2	−176.9 (3)	C17—C18—N4—Co1^i^	−2.2 (3)
C26—C21—C27—O3	−174.3 (2)	C9—C10—O1—C11	88.7 (2)
C22—C21—C27—O3	5.3 (4)	C12—C11—O1—C10	178.01 (19)
C34—C29—C30—C31	2.5 (3)	O3—C27—O2—Co1	4.4 (3)
C35—C29—C30—C31	−175.5 (2)	C21—C27—O2—Co1	−173.3 (2)
C29—C30—C31—C32	−1.4 (3)	O6—Co1—O2—C27	−16.1 (4)
C30—C31—C32—O7	177.8 (2)	O3—Co1—O2—C27	−2.70 (17)
C30—C31—C32—C33	−1.6 (3)	N1—Co1—O2—C27	−150.41 (18)
O7—C32—C33—C34	−176.2 (2)	N4^i^—Co1—O2—C27	104.81 (18)
C31—C32—C33—C34	3.3 (4)	O2—C27—O3—Co1	−5.1 (3)
C32—C33—C34—C29	−2.1 (4)	C21—C27—O3—Co1	172.6 (2)
C30—C29—C34—C33	−0.8 (3)	O6—Co1—O3—C27	178.96 (17)
C35—C29—C34—C33	177.2 (2)	N1—Co1—O3—C27	52.5 (2)
C30—C29—C35—O5	−6.7 (3)	N4^i^—Co1—O3—C27	−78.15 (18)
C34—C29—C35—O5	175.4 (2)	O2—Co1—O3—C27	2.69 (17)
C30—C29—C35—O6	172.9 (2)	C25—C24—O4—C28	−6.1 (3)
C34—C29—C35—O6	−5.0 (3)	C23—C24—O4—C28	174.7 (2)
N2—C7—N1—C6	0.4 (2)	O5—C35—O6—Co1	11.7 (3)
C8—C7—N1—C6	−177.3 (2)	C29—C35—O6—Co1	−167.95 (14)
N2—C7—N1—Co1	−178.13 (13)	O3—Co1—O6—C35	−84.34 (17)
C8—C7—N1—Co1	4.2 (3)	N1—Co1—O6—C35	60.66 (17)
C1—C6—N1—C7	−0.4 (2)	N4^i^—Co1—O6—C35	167.49 (16)
C5—C6—N1—C7	178.3 (2)	O2—Co1—O6—C35	−72.5 (3)
C1—C6—N1—Co1	178.14 (13)	C31—C32—O7—C36	−25.7 (3)
C5—C6—N1—Co1	−3.2 (3)	C33—C32—O7—C36	153.7 (2)

Symmetry codes: (i) −*x*+1, −*y*, −*z*+1.

### Hydrogen-bond geometry (Å, °)

**Table:**

*D*—H···*A*	*D*—H	H···*A*	*D*···*A*	*D*—H···*A*
C17—H015···O6^i^	0.93	2.46	3.111 (3)	127
C8—H8C···O2	0.96	2.50	3.255 (3)	136
C20—H03B···O2^i^	0.96	2.42	3.150 (3)	132

Symmetry codes: (i) −*x*+1, −*y*, −*z*+1.
